# Identification of Glycoxidative Lesion in Isolated Low-Density Lipoproteins from Diabetes Mellitus Subjects

**DOI:** 10.3390/life13101986

**Published:** 2023-09-29

**Authors:** Amjad R. Alyahyawi, Mohd Yasir Khan, Sultan Alouffi, Farah Maarfi, Rihab Akasha, Saif Khan, Zeeshan Rafi, Talal Alharazi, Uzma Shahab, Saheem Ahmad

**Affiliations:** 1 Department of Diagnostic Radiology, College of Applied Medical Science, University of Hail, Ha’il 2440, Saudi Arabia; a.alyahyawi@uoh.edu.sa; 2 Centre for Nuclear and Radiation Physics, Department of Physics, University of Surrey, Guildford GU2 7XH, UK; 3 Department of Biotechnology, SALS, Uttaranchal University, Dehradun 248011, India; maarfi.khan99@gmail.com; 4 Department of Medical Laboratory Sciences, College of Applied Medical Sciences, University of Hail, Ha’il 2440, Saudi Arabia; s.alouffi@uoh.edu.sa (S.A.); rihabakasha2000@yahoo.com (R.A.); t.alharzi@uoh.edu.sa (T.A.); 5 Department of Basic Dental and Medical Sciences, College of Dentistry, Hail University, Ha’il 2440, Saudi Arabia; saifkhan.bio@gmail.com; 6 Department of Bioengineering, Integral University, Lucknow 226026, India; zeddqazi@gmail.com; 7 Department of Biochemistry, King George Medical University, Lucknow 226026, India; uzmashahab@gmail.com

**Keywords:** advanced glycation end-products, low-density lipoprotein, immunoglobulin, lesion, methylglyoxal, type 2 diabetes mellitus

## Abstract

Methylglyoxal (MG) is a precursor for advanced glycation end-products (AGEs), which have a significant role in diabetes. The present study is designed to probe the immunological response of native and glycated low-density lipoprotein (LDL) in experimental animals. The second part of this study is to probe glycoxidative lesion detection in low-density lipoproteins (LDL) in diabetes subjects with varying disease duration. The neo-epitopes attributed to glycation-induced glycoxidative lesion of LDL in DM patients’ plasma were, analyzed by binding of native and MG-modified LDL immunized animal sera antibodies using an immunochemical assay. The plasma purified human LDL glycation with MG, which instigated modification in LDL. Further, the NewZealand-White rabbits were infused with unmodified natural LDL (N-LDL) and MG-glycatedLDL to probe its immunogenicity. The glycoxidative lesion detection in LDL of DM with disease duration (D.D.) of 5–15 years and D.D. > 15 years was found to be significantly higher as compared to normal healthy subjects (NHS) LDL. The findings support the notion that prolonged duration of diabetes can cause structural alteration in LDL protein molecules, rendering them highly immunogenic in nature. The presence of LDL lesions specific to MG-associated glycoxidation would further help in assessing the progression of diabetes mellitus.

## 1. Introduction

Diabetes mellitus is a multifactorial disease that affects macro-vasculature and micro-vasculature, including the kidney, eye, heart, and nerves. In other words, the persistency of hyperglycemic conditions is the principal cause of micro-vasculopathy but also appears to play an important role in the causation of macro-vasculopathy [1]. So, as far as we understand, diabetes is a hub of deformities related to altered metabolic states, characterized by hyperglycemia leading to impairment in protein, carbohydrate, and lipid metabolism [2]. It is already known so far that the reactive dicarbonyl and methylglyoxal (MG) formation under the effect of hyperglycemia promotes diabetes-associated complications [3]. Apart from hyperglycemic conditions, hypoxia and inflammation might also participate in the formation of methylglyoxal (MG), which promotes the glycolytic reaction. is also increased under these conditions [4]. Thus, highly reactive MG, the most potent glycating RCS, plays a vital role in the glycation of biomolecules.

**Figure 1:**
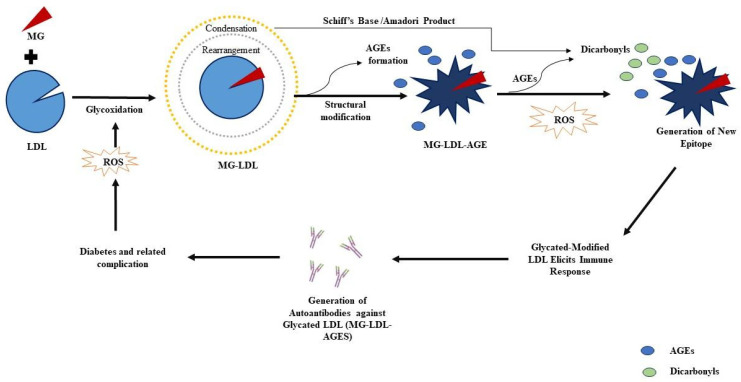
Methylglyoxal (MG)-induced glycoxidative stress and AGEs, ROS, and dicarbonyl formation may generate neo-epitopes on plasmaproteins like low-density lipoproteins (LDL), contributing to the production of autoantibodies in prolonged diabetes.

It is now well established that glycation refers to the non-enzymatic post-translational modification (NEPTM) of proteins and other biomolecules, rendering it dysfunctional. It is a concentration-dependent reaction between a free amino group of proteins and a carbonyl group of reducing sugars and other reactive carbonyl species (RCS), such as MG. The elevated level of plasma MG can react more commonly with arginine and lysine residues of proteins via complex chemical mechanisms, leading to the glycoxidation of biomolecules [5]. Consequently, glycoxidation implicates protein cross-linking, promotes AGE formation, alters the structure and function of cellular proteins, and hence dysfunctions the cellular system [6]. The variants of MG-derived AGEs have far different pathophysiological consequences, which shed light on the potential role of MG-derived AGEs in diabetes [7]. The disproportionate build-up of AGEs has been found to trigger cellular immune responses, showing AGEs as antigenic in nature; hence, the incessant theory of AGE accumulation that induces an autoimmune mechanism in a diabetic subject (Figure 1).

Dyslipidemia is a significant health problem and an important risk factor among type 2 diabetic patients [8,9]. Blood lipid levels in patients with T2DM are affected by the degree of glycemic control. T2DM patients often present with characteristic plasma lipid and lipoprotein abnormalities, including low HDL, high LDL, and elevated TC levels [10]. The high LDL level can be correlated with a lack of physical activity, genetic predisposition, and post-translational modifications. Glycation-aggregated AGEs accumulate on long-lived intima proteins, such as LDL, with aging. This process is accelerated by diabetes.High sugar and carbonyl content levels trigger a chain of LDL modifications, including oxidative and glycative modifications, enhancing the atherogenicity of the fraction [11]. The plasma concentrations of MG are elevated in diabetic individuals and are hence considered to be a significantly potent glycative agent [12]. During persistent hyperglycemia in vivo, this mechanism gets into its worst state due to the formation of reactive dicarbonyl aldehyde species such as MG, which finally leads to the onset of pathological ailments related to AGE accumulation in plasma as well as tissues [13,14,15].

Our previous study on the detection of immunogenic responses induced by glycated LDL suggested the immunogenicity of MG-glycated LDL in experimental animals [16]. Another recent investigation concluded that glycation-mediated structural perturbation of the LDL molecule might cause new antigenic determinants and LDL-AGE formation, which may be recognized as foreign molecules and can induce autoantibodies in diabetes patients [17]. The glycated LDL exhibits neo-epitopes for eliciting immune responses and generates circulating auto-antibodies in the sera of diabetes patients, showing preferential recognition of the epitopes on MG-modified LDL as linked to their native analogue. Although MG-modified LDL is pro-inflammatory in nature and induces autoimmune responses in diabetic patients. This might be possible due to glycation-mediated structural lesions on LDL, which are recognized as a whole new form of LDL as a foreign molecule by the diabetic subject’s immune system. However, no study or investigation conveyed the glycative or glycoxidative lesion detection of LDL in type 2 diabetes mellitus (DM) subjects.An imminent and epi-centroidal study is demanded to demonstrate the glycoxidative lesion detection on plasma LDL of diabetes-afflicted subjects with varying disease duration, which might serve as a promising marker for diabetes and its associated complications.

This study is designed to investigate the affinity of LDL isolated from the plasma of type-2 diabetes mellitussubjects tested with sera anti-MG-LDL antibodies from experimental animals immunized with MG-LDL. The antibodies were raised against MG-LDL in NZW female rabbits, which preferentially binds with more affinity to MG-LDL than native-LDL (N-LDL). By using these antibodies, we can examine whether the plasma levels of MG-LDL are elevated in DM subjects. Therefore, this study looked into prolonged exposure tohyperglycemia-related LDL modifications, including MG-LDL, and their role in the development of an immunogenic response in diabetes.

## 2. Results

### 2.1. Modification Detection in Glycated LDL

**Figure 2:**
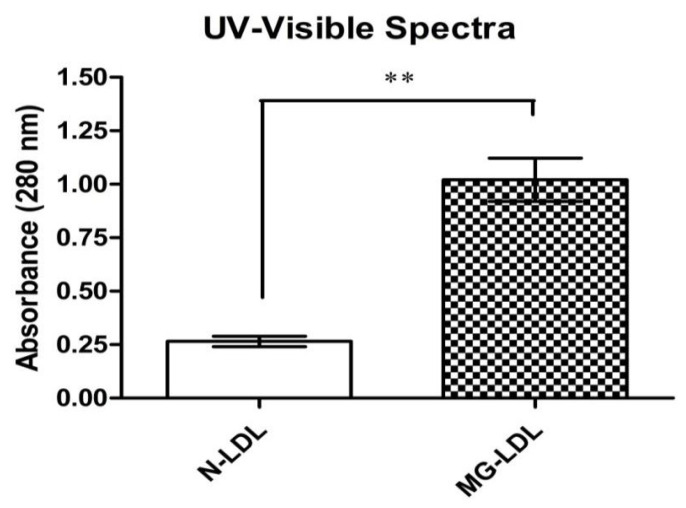
Characterization with UV-Vis spectra of unmodified naturalLDL (□) and LDL modified with methylglyoxal (MG) (_▓_)_;_ incubation time was 10 days. The data are mean ± SD of three independent experiments. The level of significance for N-LDL v/s 5 mM of MG-LDL on fifth day of glycation reaction was ** *p* < 0.001.

The characterization outlineforunmodified natural LDL (N-LDL) and MG-glycatedLDL (MG-LDL) at 280 nm was performed by UV-Visible spectroscopy. The UV-Vis absorbance peak at 280 nm showed a hyperchromicity increase upon modification of LDL by MG. In comparison to N-LDL, the hyperchromicity of MG-LDL increased by 65% (max.) with 5 mM MG (Figure 2).

### 2.2. Biochemical Estimation-Ketoamines, HMF, Protein-Carbonyl, and CML in Sera Samples

**Figure 3:**
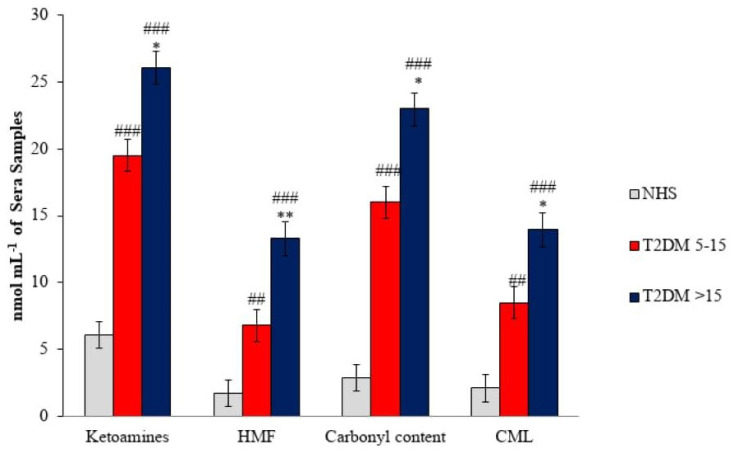
The bar diagram represents the estimation of ketoamines, HMF, Carbonyl content, and CML-AGE in T2DM (80-D.D. 5–15 years and 50-D.D. > 15 years) serum samples with normal healthy subjects (NHS, *n* = 50). Values are expressed as the mean ± SD of three independent experiments. The values were significantly different from NHS sera at ^###^ *p* < 0.0001. The values were significantly different from NHS sera at ^##^ *p* < 0.001. The values are significantly different from T2DM (D.D. 5–15) patients’ sera at * *p* < 0.01. Significantly different from T2DM (D.D. 5–15) at ** *p* < 0.001.

The calorimetric NBT assay, with the help of UV-Visible spectroscopy, was performed in order to quantify the early glycation products (EGPs), that is, ketoamine compounds, in the sera of diabetic patients as compared to normal healthy subjects (NHS) sera. The normal human serum (NHS)group (*n* = 50) exhibited a mean ketoamine of 5.9 ± 0.87 nmol mL^−1^ sera, while the patient group had significantlyenhancedketoamine as compared to NHS sera (Figure 3). The above increase in ketoamine is found to be 19.5 ± 3.5 nmol mL^−1^ in DM (D.D. 5–15, *n* = 80) and 26.1 ± 12.13 nmol mL^−1^ in DM (D.D. > 15, *n* = 50), respectively.

In the NHS sera, the amount of HMF content was found to be near to negligible, i.e., 1.7 ± 1.03 nmol mL^−1^. However, the amount of HMF significantly increased in DM (D.D. 5–15) sera; the HMF content was 6.8 ± 1.7 nmol mL^−1^, whereas in DM (D.D. > 15, *n* = 50) patients’ sera, it was found to be 13.34 ± 2.1 nmol mL^−1^. The bar diagram expresses the significance of the HMF content of the NHS and both groups of diabetes subjects’ sera, shown in Figure 3.

In the investigation of protein-carbonyl moieties, both patient groups (DM with D.D. 5–15 and DM subjects with D.D. > 15) also showed a significantly higher amount of carbonyl content than NHS sera samples. The mean values of estimated carbonyl contents (±SD) from three independent assays of serum samples from DM (D.D. 5–15, *n* = 80) and DM (D.D. > 15, *n* = 50) patients were 16 ± 1.9 nmol mL^−1^ and 23 ± 2.3 nmol mL^−1^ sera. DM (D.D. > 15) patients showed a significantly higher protein-carbonyl content level than D.D. 5–15 and was highly significant compared to NHS (*n* = 50) (Figure 3). NHS group sera showed considerably less carbonyl content (2.9 ± 1.1 nmol mL^−1^ sera). The significant increase in carbonyl content corresponded to a nearly four- to five-fold increase in the protein-carbonyl content in the sera of diabetic patient groups as compared to the NHS sera.

The non-fluorogenic CML AGEs were quantified in the sera of NHS subjects and diabetic patients by a CML detection ELISA kit. The mean level of CML content in the groups DM (D.D. 5–15) and DM (D.D. > 15) was 14 ± 2 and 8 ± 1.5 nmol mL^−1^ serum, respectively, while that in the NHS group was 1.8 ± 1 nmol mL^−1^ serum (Figure 3). The CML quantification results show an increase in CML-AGEs due to prolonged disease duration in DM patients.

### 2.3. Characterization of Purified IgG from Immunized Animals

**Figure 4:**
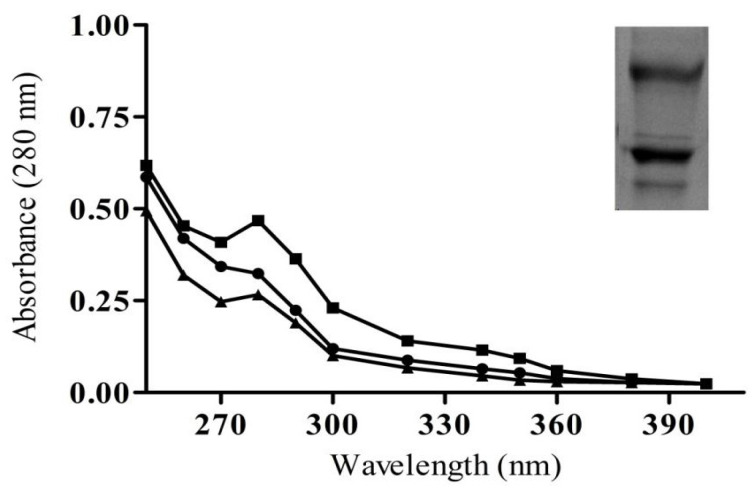
Direct binding ELISA profile of IgG purified from immunized animal sera: The graph represents the elution profile of IgG isolated from pre-immune sera (▲), native-LDL (N-LDL) (●) and MG-glycated LDL (MG-LDL) (■) immunized NZW female rabbits’ sera. The spectra are the average of three determinations. Insert: SDS-PAGE image showing bands of isolated IgG on an 8% polyacrylamide gel.

An agarose-packed column was used as an affinity column assay to isolate and purify immunoglobulin G (IgG) from N-LDL and MG-LDL animal immunized sera (NZW female rabbits). The homogeneity of eluted IgG was confirmed by a symmetric peak, suggesting an increase in concentration of IgG with an increase in absorbance at 280 nm (Figure 4). Further, the quality of the obtained IgG was analyzed by running an 8% non-reducing SDS-PAGE. The graph of homogeneity is shown in Figure 4.

### 2.4. Binding Affinity of IgG from Experimental Animals towards Patients LDL

**Figure 5:**
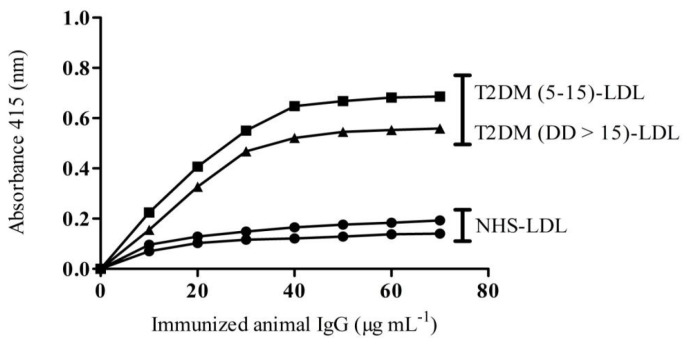
The isolated IgG saturation with LDL isolated from NHS and diabetic patients: The graph represents the saturation of anti-native LDL IgG (N-LDL-IgG) and anti-MG-glycated LDL IgG (MG-LDL-IgG) with LDL purified from plasma of NHS (●) and T2DM subjects with both disease durations, i.e., D.D. 5–15 (▲) and D.D. > 15 (■). The increase in absorbance was observed at 415 nm. The microtiter wells were coated with 10 μg mL^−1^ of LDL from NHS and T2DM subjects. The datarepresented in the figure arefrom three independent experiments.

ELISA-direct binding of native LDL depicts a low immunogenic response and therefore shows an average titer of antibodies (>1:1600). However, MG-LDL immunized animal sera showed high titer antibodies (>1:25,600) and were found to be much more immunogenic compared with pre-immune and LDL (native form) immunized animal sera, as detailed in our previous animal immunization study (Khan et al., 2018 [16]). On the basis of previous binding affinity results, the binding affinity of the purified IgG from immunized animal sera was used to check the antigen (LDL) amount required for saturation isolated from NHS-LDL and DMD.D. 5–15-LDL and D.D. > 15-LDL. The animals immunized with native-LDL (N-LDL) isolated antibodies, anti-N-LDL antibodies (N-LDL-IgG), showed a saturation level of 20 μgmL^−1^ with LDL of normal healthy subjects (NHS-LDL), whereas anti-MG-LDL antibodies (MG-LDL-IgG) from animals immunized with MG-LDL showed a saturation level of 40 μg mL^−1^ with LDL of DM patients (D.D. 5–15-LDL and D.D. > 15-LDL) (Figure 5). The IgG molecules that were purified from pre-immune sera did not show significant binding, as shown in our previous study. Binding of the N-LDL-IgG from immunized animals with LDL isolated from normal healthy subjects (NHS-LDL) was of low magnitude. However, the MG-LDL-IgG showed significant binding towards the D.D. 5–15-LDL and D.D. > 15-LDL.

### 2.5. Glycoxidative lesion Detection by Inhibition ELISA

**Figure 6:**
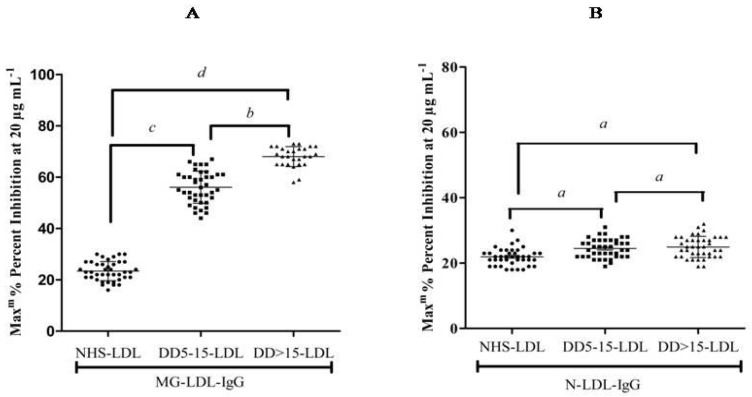
Competitive inhibition ELISA: The purified IgG samples of native and MG-LDL-immunized animals showed significant binding affinity towards purified LDL samples of T2DM (D.D. > 15) patients and T2DM (D.D. 5–15) patients as compared to NHS-LDL. The microtiter plates were pre-coated with native and MG-glycated LDL (10 μg mL^−1^). (**A**) The significant % inhibition shown at higher inhibitory concentration (20 μg/mL) was observed with purified LDL of patients (T2DM: D.D. > 15 and D.D. 5–15) as compared to NHS-LDL. Significantly different from NHS-LDL at ^b^ *p* < 0.05. Significantly different from NHS-LDL at ^c^ *p* < 0.005. Significantly different from NHS-LDL at ^d^ *p* < 0.001. (**B**) No significant % inhibition of N-LDL-IgG binding was shown with purified LDL of patients (T2DM: D.D. > 15 and D.D. 5–15) as compared to NHS-LDL. Non-significant from NHS-LDL at ^a^ *p* > 0.05. Data are presented as mean ± S.D. of three independent experiments.

This experiment was carried out to analyze specifically the positive LDL (MG-glycated-modified LDL) samples from the plasma of diabetic subjects with different disease durations (DMD.D. 5–15 and D.D. > 15). The glycative lesion detection was performed on a total of 180 plasma-isolated LDL samples. Out of which, LDL molecules were purified from DM patients (80 samples) with a disease duration of 5–15 years (D.D. 5–15), 50 samples of DM patients with a disease duration of more than 15 years (D.D. > 15), and 50 normal healthy subjects (NHS). The isolated LDL from DMD.D. 5–15-LDL and D.D. > 15-LDL patients were used as inhibitors. The N-LDL and MG-LDL-coated wells of microtiter plates were used to estimate the glycative lesion in LDL from DMD.D. 5–15-LDL and D.D. > 15-LDL patients’ plasma samples. The LDL from DMD.D. 5–15-LDL and D.D. > 15-LDL patients showed significant binding with IgG of MG-LDL (MG-LDL-IgG) immunized rabbits as compared to N-LDL-IgG immunized animals. The maximum and significant inhibition was found at 20 μg mL^−1^ of DMD.D. 5–15-LDL (*p* < 0.005)and D.D.> 15-LDL (*p* < 0.001) with MG-LDL-IgG, whereas the maximum inhibition of 22% was observed at 20 μg mL^−1^ of NHS-LDL with MG-LDL-IgG (Figure 6A).However, N-LDL-IgG binds with coated N-LDL, referring to the slight or non-significant inhibition in the range of 20–25% by NHS-LDL, D.D. 5–15-LDL and D.D. > 15-LDL (*p* > 0.05) (Figure 6B).

The maximum inhibition was shown by LDL from DMD.D. > 15-LDL (64%), which is greater than the inhibition exhibited by LDL from DMD.D. 5–15-LDL (55%), inhibited binding of MG-LDL-IgG to its specific immunogen MG-LDL (invitro glycated LDL) to a significant level.

## 3. Discussion

The structural alteration induced by NEPTMs makes biomolecules highly immunogenic, recognizesthem as foreign molecules, and elicits the formation of autoantibodies. Several reports on the glycation-mediated immunological response suggest the significant role of in vivo AGEs that function as immunological neo-epitopes, which further induce auto-antibodies that promote the progression of immunological complexities in diabetes mellitus and its mediated comorbidities [18,19].

Biophysical and biochemical characterization unveils the overall LDL structural alteration in glycoxidatively modified conditions [20]. The UV-Vis spectroscopy assay indicated the exposed chromophoric aromatic LDL amino acid residues might be due to the folding and unfolding or fragmentation of protein residues and AGES formation. The glycation mechanism leads to structural perturbation of proteins and promotes a transition in the level of native LDL epitopes into immunogenic neo-epitopes. This transition indicates a direct relationship between glycation and immunogenicity at anin vivo level. Several reports have already proven the accountability of antibodies in the progression of several pathological disorders, such as diabetes-associated comorbidities [19,21].

The antigenicity of MG-modified LDL was evaluated by an immunological study in experimental rabbits. However, in the present study, glycation or glycoxidative lesions of plasma LDL were detected in DM patients with both disease durations (D.D. 5–15 and D.D.> 15). The immunological properties of LDL isolated from DM patients were detected by probing antibodies (IgG) that are raised against native and glycated LDL (MG-LDL) in NZW rabbits [16]. The in vitro dicarbonyl-mediated LDL glycation results in secondary and tertiary structural and functional alteration of the LDL molecule, similar to previously discussed LDL glycation [22,23]. The overall changes in protein structure can make the molecule more immunogenic than its native form, which would induce auto-antibodies against several AGE-protein structures and have also been reported in the sera of diabetic patients, triggering an autoimmune response [18].

In the confirmation of alterations in DM patients LDL via methylglyoxal-mediated glycation, in the direct binding ELISA studies, DM patients LDL was observed to exhibit high specificity compared to its NHS counterpart, demonstrating the presence of neo-epitopes on LDL. In direct binding ELISA, the antigenic specificity of affinity purified MG-LDL-IgG elicits the prevalence of antibodies that preferentially recognize the modified epitopes of LDL isolated from DM patients. In inhibition ELISA studies, DMD.D. > 15 and D.D. 5–15 patients plasma isolated LDL exhibited strong binding towards the MG-LDL-IgG generated in the rabbits, demonstrating that MG-modified LDL has generated a specific immune response against the glycated LDL. The higher and more specific recognition of MG-LDL in serum IgGs might be due to the increased age of diabetes patients (DD > 15 years), which concomitantly exaggerated protein oxidation that induced serum albumin structural alterations, thus generating unique epitopes that might play a role in the production of MG-LDL-Antibodies. MG-AGEs were able to discriminate which individuals with autoantibodies would progress at a faster rate to later-stage diabetes, providing a potential new clinical biomarker for determining the rate of disease progression and pointing to contributing metabolic pathways [24].

From our results, it has been ascertained that individuals with a longer duration of diabetes exhibited significantly higher amounts of serum carbonyl content like methylglyoxal and AGEs as compared to age-matched non-diabetic subjects. Autoantibodies against glycated LDL levels were also analyzed by AGEs-specific fluorescence and ELISA-based detection of serum antibodies [17]. Our findings help us understand the role of glycation in clinical conditions, the accumulation of MG, and its impact on mediating glycoxidative lesions on LDL molecules recognized as antigens by their specific raised antibodies. Thus, we can draw the conclusion that this study would be helpful to explore the findings of glycoxidative lesion detection in diabetes patients, which might uncover the MG-glycated LDL role, to deal with secondary complications associated with diabetes.

## 4. Materials and Methods

### 4.1. Ethical Statement

Procedure and protocol with approval number: IEC approval number: IEC/IIMSR/2017/39 has been obtained from the Institutional Animal Ethical Committee Centre (IAECC) at Integral University (IU), Lucknow, India, to perform the animal immunization protocol schedule for New Zealand White (NZW) female rabbits. A total of 12 rabbits (family Leporidae) were accommodated and handled in a careful and kind manner in the animal house amenity of Integral University, catering to 12 to12 h of bright light and darkness cycles in an aerated expanse with an 80 °F temperature and 50% humidity. Acclimatization was performed for three weeks by providing these animals with a proper diet and water bottles.

The Institutional Ethics Committee (IEC), with the consensus of the Integral Institute of Medical Sciences and Research (IIMS&R), sanctioned the glycoxidative lesion detection protocol and provided the approval number. IEC/IIMS&R/2017/39. The patients suffering from type 2 diabetes mellitus (DM) with a disease duration of 5–15 years (D.D. 5–15) and more than 15 years (D.D. > 15) who attended the OPD/IPD in IIMS&R, Lucknow, India, were selected for this study.

### 4.2. Precipitation of LDL from Human Plasma

Human plasma was recovered from blood samples of normal healthy subjects (NHS) and DM (D.D. 5–15) and DM (D.D. > 15) patients. The citrate-heparin buffer is used for low-density lipoprotein (LDL) precipitation, as described by Wieland and Seidel (1989). Further, purified LDL was quantified with the bicinchoninic acid assay (BCA) method described previously [25,26]. The precipitated LDL samples were kept at −20 °C for storage.

### 4.3. Glycation of LDL

The glycation of LDL with methylglyoxal (MG) was performed using a previously standardized protocol from our laboratory, in which we established the minimal concentration for the glycation of LDL [27]. Briefly, plasma-isolated LDL from NHS (70 μg mL^−1^) was glycated with 5 mM of MG in 100 mM PBS of 7.4-pH along with 50 μL of 0.05% sodiumazide as an antimicrobial agent. The reaction mixtures were incubated at 37 °C for 10 days under sterile conditions. Native LDL (N-LDL) was considered a control sample.

### 4.4. Early Glycation Detection-Nitroblue Tetrazolium (NBT) Assay

To detect the ketoamines, an NBT reduction assay was performed with the sera of NHS and DM patients. The NBT assay could imply discernment between NHS and diabetic subjects on the basis of early glycation products. The isolated serum samples (20 μL) from blood were mixed with 180 μL of freshly prepared 250 μM NBT solution in Na_2_CO_3_/NaHCO_3_ buffer (1000 mM bicarbonate buffer of pH 10.8). The nextstep is to incubate the sample mixtures at physiological temperature, which is 37 °C, until the yellow mixture changes to a purple mixture. The absorbance for ketoamine detection was recorded at 525 nm wavelength, and the molar extinction coefficient of 12,640 M^−1^ cm^−1^ was used to calculate ketoamine concentration (nmol mL^−1^) [28]. 

### 4.5. Hydroxymethylfurfural (HMF) Estimation

Hydroxymethylfurfural (HMF), an organic product of glycation-mediated reducing sugar dehydration, can be detected by thiobarbituric acid (TBA) [29]. The freshly separated blood sera (0.1 mL) of NHS and diabetic patients was mixed with 0.1 mL of 1 M oxalic acid and left for 1 h in the water bath. After cooling to room temperature, trichloroacetic acid (TCA) (40%) was mixed in the solutions. TBA was addedto the supernatant taken from the solution mixture and incubated at 37 °C for 30–40 min. After the appearance of color, HMF concentration (nmol mL^−1^) was evaluated by using the molar extinction coefficient of 4 × 104 M^−1^ cm^−1^ at 443 nm wavelength.

### 4.6. Glycation Intermediates-Protein Carbonyl Estimation

Carbonyl group is a well-established marker of oxidative stress [30]. However, it is also used for glycation of protein as preliminary biomarker. Protein glycation-induced carbonyls were estimated in the sera of NHS and diabetic subjects by using 2,4-dinitrophenylhydrazine (DNPH). A small volume of serum (0.1 mL) from NHS and diabetic subjects was taken and mixed with 0.5 mL of 10 mM DNPH in 2 N of HCL. This mixture for carbonyl estimation was incubated at room temperature (RT) for the duration of 1 h (1 h). Following incubation, 0.5 mL volume of TCA (20% v/v) was added, and the mixture was centrifuged at 10,000 rpm for 10 min to precipitate DNPH. The precipitated pellet was washed three times with 0.5 mL of ethanol-ethyl acetate solution (1:1 v/v) to relieve unbound DNPH. The precipitated pellets were dissolved in 0.25 mL of guanidinium-HCL solution (6 M). Protein carbonyl concentration (nmol mL^−1^) was evaluated by using a molar extinction coefficient of 22,000 M^−1^ cm^−1^ at 370 nm wavelength.

### 4.7. Evaluation of Nonfluorogenic CML-AGE

Carboxymethyllysine (CML) is well known as a nonfluorogenic AGE and is detected in the sera of the NHS and DMD.D. 5–15 and D.D. > 15 patients by using a sandwich ELISA kit from Bioassay Technology Laboratory (Korain Biotech Co., Ltd., London, UK) as previously described [31].

### 4.8. Preparation of Anti-MG-LDL Antibodies (MG-LDL-IgG)

Native and MG-glycated LDL (MG-LDL) were prepared for immunization. The reaction mixture of MG with LDL was used as an antigen for the immunization of NZW female rabbits (*n* = 6). Native LDL antigen was used as a control for the immunization of NZW female rabbits (*n* = 6), and the procedure was followed as described in our previously reported immunization study [17]. Ten days after the final dose, antisera were tested with MG-LDL by direct binding ELISA in our previous immunization study [16]. The antisera of rabbits with the highest titerwere used for anti-MG-LDL antibodies (MG-LDL-IgG) purification for further investigations.

### 4.9. Direct Binding ELISA

To detect the binding affinity and reactivity of animal sera purified IgG with precipitated human LDL from DM patients and NHS plasma samples, direct binding ELISA was performed. Polystyrene (96-well) microplates were coated with the LDL from NHS and DM patients (100 μg mL^−1^), and then increasing purified IgG concentrations (10–70 μg) were applied as previously described [17].

### 4.10. Inhibition ELISA-Glycative Lesion Detection

An inhibition or competitive inhibition ELISA assay was performed to ensure the binding specificity of raised antibodies towards the antigen [32]. The protocol assists in evaluating the specificity of isolated IgG from native and glycated LDL immunized animal sera by binding with LDL of DM (D.D. 5–15-LDL and D.D. >15-LDL) patients and NHS (NHS-LDL). The microtiter-ELISA plates coated with 0.1 mL(10 μg mL^−1^) of native-LDL (N-LDL) and MG-LDL were incubated for 2.5 h at room temperature (RT), followed by overnight incubation of the plates at 4 °C. A constant amount of N-LDL-IgG and MG-LDL-IgG (showed significantly higher binding affinity than N-LDL-IgG) from immunized animals was mixed with a different concentration of LDL isolated from the NHS and diabetic subjects (LDL; 0–20 μg mL^−1^ from the NHS, D.D. 5–15-LDL, and D.D. >15-LDL). The prepared mixture was incubated at RT for 2.5 h, followed by an overnight incubation at 4 °C. The incubation mixture consists of antigen-antibody immune complexes that are coated in the uppermost layer of wells instead of antibodies alone. Further methods and steps are the same as those employed in the protocol for direct binding ELISA. The inhibition percent of IgG was calculated by using the formula:

Percent IgG inhibition = 1 − (A inhibited/A uninhibited) × 100

### 4.11. Statistical Analysis

The sample analysis for biochemical and immunological assays was taken in triplicate for all the measurements, and the data were represented as mean ± SD. The statistical significance was evaluated by the ANOVA method through GraphPad Prism version 5.01 for Windows (GraphPad Software, San Diego, CA, USA).

## 5. Conclusions

Increase in plasma MG concentration mediates a glycoxidative lesion of native LDL and causes the accumulation of MG-LDL in plasma.Glycoxidation of LDL molecules in hydroxy-methyl furfural, carbonyl content, ketoamines, MG-LDL-AGES, and CML, with an increase in the disease duration of DM. The detection of circulating glycated LDL in the DM patient’s plasma showed significant specificity towards MG-modified LDL-specific IgG isolated from native and glycated immunized experimental animals. This brings us to the conclusion that structural modification of LDL causes LDL Lesionsand induces neoepitopes on LDL due to prolonged exposure to MG, which has a strong role in increasing circulating autoantibodies in individuals with diabetic pathophysiology. Hence, such prolonged durations of diabetes can make individuals immunologically imbalanced, and this might play a role in associated complications of diabetes.

## Data Availability

Data are available upon request from the corresponding author.
